# Layout optimization of multi-level cold chain storage facilities in agricultural producing areas considering type and capacity constraints

**DOI:** 10.1371/journal.pone.0313062

**Published:** 2025-02-11

**Authors:** Qian Huang, Guijun Zheng, Shuangli Pan, Huiyu Liao, Zehua Jiang

**Affiliations:** 1 School of Logistics, Central South University of Forestry and Technology, Changsha, Hunan, China; 2 Business School, Central South University of Forestry and Technology, Changsha, Hunan, China; 3 Hunan Key Laboratory of Intelligent Logistics Technology, Changsha, Hunan, China; University of Shanghai for Science and Technology, CHINA

## Abstract

The effective circulation of fresh agricultural products is conducive to increasing farmers’ income and improving the living standards of urban residents. Cold chain storage facilities in agricultural producing areas play an important role in ensuring the quality of agricultural products, extending the freshness period of goods, and improving logistics efficiency. Different types of fresh produce have different requirements for refrigeration and often require transshipment due to quantity constraints. In addition, there are economies of scale in the construction and operation of cold chain storage facilities. Based on the above considerations, with the aim of minimizing the total daily cost, an optimization model for the layout of multi-level cold chain storage facilities is established to determine the number, location, type and capacity of cold chain storage facilities at the same time. Genetic algorithm is chosen to solve the model according to the characteristics of the model. Taking J County of China as an example, the model is proved to have strong operability and applicability. It is of guiding significance and reference value to optimize the layout of cold chain storage facilities in rural areas.

## 1 Introduction

With the continuous advancement of agricultural modernization and the increasing requirements of consumers for the quality of agricultural products, the demand for the cold chain of agricultural products is becoming more and more vigorous. Logistics in agricultural producing areas is a key part of the cold chain. If there are problems such as damage to the freshness of agricultural products at the beginning, subsequent work will be futile. Cold chain storage facilities allow products to be kept in a low temperature environment after picking, which can effectively ensure quality and reduce loss. Meanwhile, it is also conducive to regulating the transportation activities in rural areas and improving the efficiency of agricultural products circulation.

However, the construction of cold chain storage facilities is difficult to make decisions. In order to better serve the storage and transportation of agricultural products, it is not only necessary to reasonably determine the number and location of storage facilities, but also to consider the type of facilities to adapt to the cold storage needs of different agricultural products. At the same time, the construction and operating costs of cold chain storage facilities are high, and it is important to reasonably determine the scale of construction. In addition, because the amount of agricultural products produced and sold in one place is often limited, many times it is necessary to transfer, which will involve different levels of cold chain storage facilities. Therefore, the rational layout of cold chain storage facilities in agricultural producing areas is worthy of in-depth discussion.

## 2 Literature review

Logistics facility layout has always been one of the hot spots in academic research. With the rapid development of the cold chain of agricultural products, the layout of cold chain storage facilities has also attracted much attention, but the existing research mainly focuses on the distribution center layout of the“last mile” of the cold chain. Dou et al. [1] established an optimization model for location selection of cold chain logistics distribution centers with the goal of minimizing total cost, considering the perishable characteristics of refrigerated food. Singh et al. [2] established a mixed integer linear programming model considering the loss of product value during cold chain transportation, which considered the site-allocation problem of cold chain storage facilities with service distance constraints. Zhang et al. [3] took the transfer station and distribution center of agricultural products as a two-level logistics storage facility and found the best way of layout by establishing a model. Merak et al. [4] built a mathematical location model to obtain the optimal location of agricultural products logistics center.

There are relatively few researches on the layout of cold chain storage facilities in agricultural producing areas. Yang et al. [5] established a cold chain distribution center location optimization model based on freshness, considering the cold chain distribution network of pre-cooling station → distribution center → demand point. Ma et al. [6] considered the loss of various types of fresh agricultural products in the logistics process from producing area to pre-cooling station to logistics center, and established a mixed integer linear programming model. Li et al. [7] studied the optimal layout scheme of rural cold chain storage facilities. Liang et al. [8] built a cold storage location model of producing area with the minimum total cost as the target considering the satisfaction of farmers. The existing studies mainly consider quantity and location decision, except for reference 6, which basically does not consider the difference in cold storage demand of different types of fresh agricultural products, and does not involve the type decision of cold chain storage facilities in agricultural producing areas.

Depending on whether the capacity limit of the facility is considered, facility location problem (FLP) is divided into uncapacitated facility location problem (UFLP) [9,10] and capacitated facility location problem (CFLP) [11–13]. However, the current studies on facility location basically assume that facility scale is unlimited [14,15], and facility construction cost is fixed or a non-linear function of facility construction quantity, without considering the scale economy effect in facility construction [16]. In fact, the scale of construction will significantly affect the construction and operation costs of facilities, especially for cold chain storage facilities. Due to the high construction and operation costs, it is necessary to improve storage rates and refrigeration efficiency through scientific and reasonable scale planning.

As for the layout of multilevel storage facilities, most of the existing studies focus on industrial products, and there are not many studies on agricultural products. Arturo et al. [17] studied the location of logistics storage for perishable products in mountainous areas based on multi-level and multi-product transportation needs. Gharaei et al. [18] established a multi-product, multi-wholesale, multi-level and integrated logistics warehousing model under the situation of shortage and limited warehouse space. He et al. [19] established a rural three-level logistics node layout model aiming at minimizing operating costs according to the actual situation in China.

To sum up, there are many research results on the construction of cold chain storage facilities for agricultural products, but the focus of attention is different. The comparison of existing studies is shown in Table 1, depending on whether they are specific to first mile logistics, and whether refrigeration demand, capacity limitation, and multi-level storage facilities are considered. It can be seen that there are not many studies on the construction of cold chain storage facilities that focus on the “first mile” of agricultural products, and few studies consider the refrigeration demand of products and capacity limitation. The construction of multi-level storage facilities has attracted attention, but there are not many studies on the origin logistics of agricultural products. Studies that consider all four factors at the same time are lacking.

**Table 1:** Comparison of existing studies on the construction of cold chain storage facilities for agricultural products.

Document number	First mile logistics	Refrigeration demand	Capacity limitation	Multi-level storage facilities
1,2,4				
3				√
5,7,8	√			
6	√	√	√	
9–10, 14–16				
11–13			√	
17,18				√
19	√			√

Note: A “√” indicates that this factor was taken into account in the relevant literature.

In view of this, this paper intends to study the layout optimization of multi-level cold chain storage facilities in agricultural producing areas considering type and capacity constraints, in order to find the optimal layout scheme with the lowest total cost. Different from previous studies, this study has the following main features:

1. Constructing multi-level cold chain storage facilities for fresh agricultural producing areas.
2. Consider adopting appropriate refrigeration methods for various fresh agricultural products.
3. Consider that different types and capacities of cold chain facilities have corresponding construction and operational costs.
4. While determining the number and location of cold chain storage facilities, it also determines their type and capacity.

## 3 Method

### 3.1 Problem description

The logistics of agricultural products in their producing areas mainly refers to the logistics activities generated during the process of agricultural products’ transportation from villages to cities. Taking the administrative pattern of “county, township, and village” in China as an example, it mainly refers to the “upward” logistics activities of agricultural products generated within the county area. This paper takes China as an example to study how to reasonably layout cold chain storage facilities for agricultural products within county boundaries, in order to improve the quality and efficiency of agricultural product circulation.

**Fig 1:**
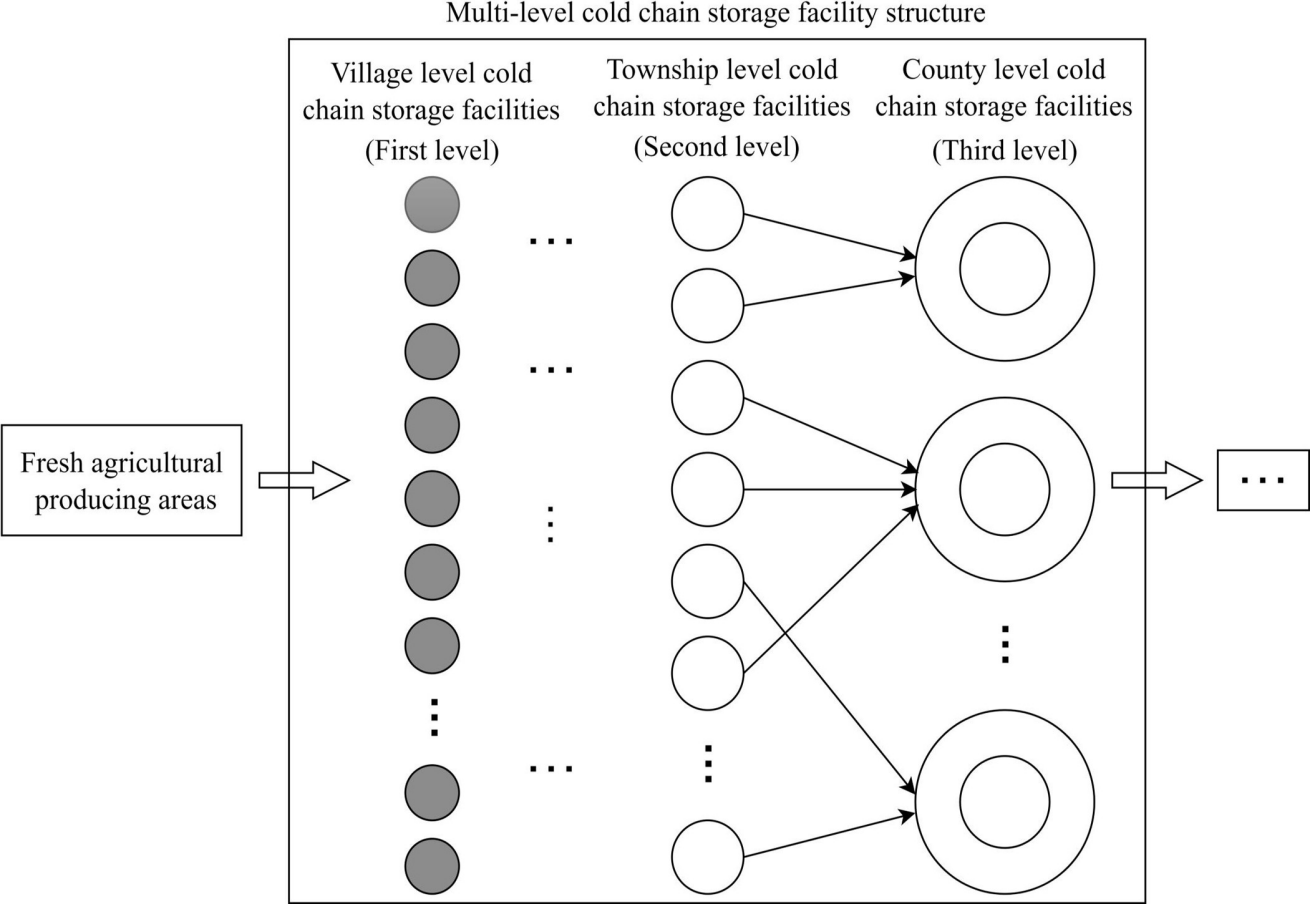
Structural diagram of multi-level cold chain storage facilities in agricultural producing areas (taking China as an example).

According to China’s “county, township and village” logistics development policy, this paper sets the cold chain storage facilities of agricultural produce areas as three levels. The first level is the village level, and the facilities are mainly pre-cooling warehouses set up in the fields. The second level is the township level, and cold chain storage facilities are responsible for collecting agricultural products in the township or adjacent villages. The third level belongs to the county level, the storage scale is large, many times only one, and it is an important node connecting sales points outside the county. The structure of multi-level cold chain storage facilities in agricultural producing areas is shown in Fig 1.

In view of the diversity of fresh agricultural products, there are also different types of cold chain storage facilities. According to the visits to several rural areas in southern and northern China, combined with the research results of Shi et al. [20] and He [21], the common types of cold storage for different fruits and vegetables are shown in Table 2. Therefore, when arranging cold chain storage facilities in agricultural producing areas, it is necessary to choose the appropriate type of cold storage facilities according to the needs of local agricultural products. Overall, it is necessary to determine the type, quantity, location and capacity of cold chain storage facilities according to the production and sales of local agricultural products, so as to obtain the optimal layout scheme.

**Table 2:** Common types of cold storage for different fruits and vegetables.

Cold storage type	Applicable type	Characteristics
Ventilated cold storage	Root vegetables	Not particularly strict on temperature.
Air-cooled cold storage	Leafy vegetables	High requirements on temperature.
Water-cooled cold storage	Fruit vegetables	Very strict on temperature.
Air regulated cold storage	Fruits	High requirements on gas concentration.

### 3.2 Model assumptions

**Fig 2:**
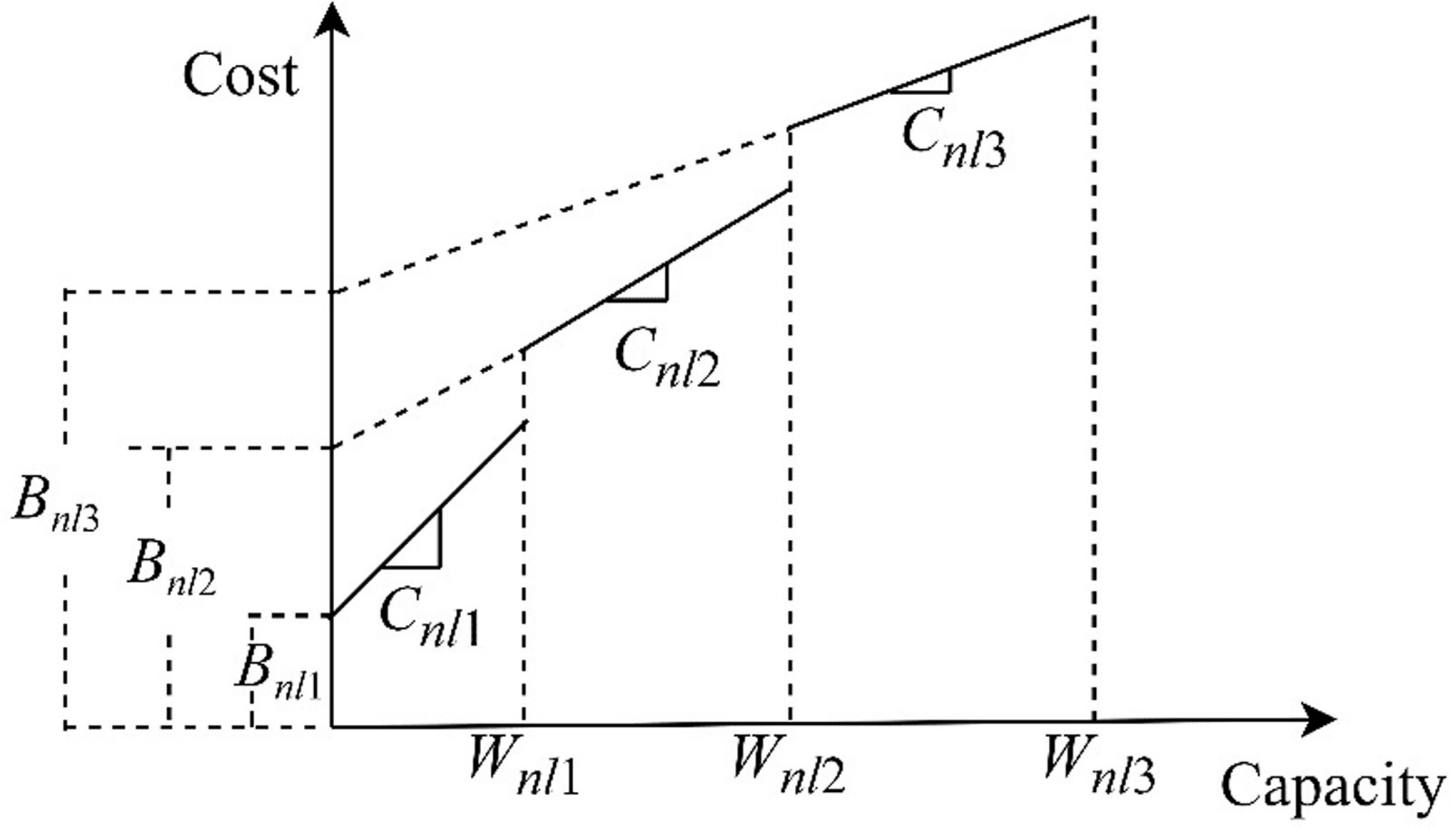
Cost function of storage facility construction.

1. At most one cold chain storage facility can be built in each candidate site.
2. Each alternative node is familiar with the local transportation routes, and the product transportation routes are all optimal.
3. Cross level transportation of goods is not accepted during the transportation process.
4. The transportation demand for fresh agricultural products at all levels of cold chain storage facilities must be met and can only be fulfilled by one upper level node.
5. The loss cost of fresh agricultural products after harvesting increases with the increase of time *t*. Using the exponential penalty cost function *F(t)* = *α*(*e*^*βt*^-1) proposed by Fujiwara et al. [22] to describe the cost of fresh agricultural products during transportation due to freshness loss. If $F(t)=\alpha_{1}(e^{\beta_{1}t}-1)$ and $F(t)=\alpha_{1}(e^{\beta_{1}t}-1)$ are the loss cost functions from the first level node to the second level node and from the second level node to the third level node, respectively, then the loss cost coefficients are expressed as: *α*_1_ > *α*_2_ >0, *β*_1_ > *β*_2_>0.
6. According to Wu et al. [16], both fixed cost *B*_*nlh*_ and variable cost *C*_*nlh*_ of storage facility construction are related to capacity, as shown in Fig 2. In addition, operating cost *O*_*nlh*_ is also related to capacity. The larger the scale of warehouse construction, the higher the fixed construction cost, and the lower the unit variable construction cost and operating cost.

The parameters and variables used in the modeling process of this paper involve total daily cost, node level, node type set, node capacity level set, etc. The details are in the appendix.

### 3.3 Model establishment

#### 3.3.1 Target analysis

The goal of optimizing the layout of multi-level cold chain storage facilities in agricultural producing areas considering type and capacity limitations is to minimize the daily total cost of the system, including the construction and operation costs of cold chain storage facilities, as well as the transportation and loss costs of fresh agricultural products.

1. Construction cost

The construction cost *Z*_*1*_ of cold chain storage facilities is related to the location, type, and capacity, including fixed construction costs and variable construction costs related to construction capacity.


$$Z_{1}=\sum_{i=1}^{P_{n}}\sum_{n=2}^{N}\sum_{l\in L}\sum_{h\in H}X_{nlh}^{i}\;B_{nlh}^{i}+\sum_{i=1}^{P_{n}}\sum_{n=2}^{N}\sum_{l\in L}\sum_{h\in H}C_{nlh}^{i}\;Cap_{nl\hbar }^{i}$$ (1)

2. Operating cost

First level cold chain storage facilities are often built in the field, and their operating costs are mainly related to the volume of product to be circulated. The operating cost *Z*_*2*_ of the first level cold chain storage facility is:

$$Z_{2}=\sum_{i=1}^{P_{1}}\lambda \;{\text{M}}_{1}^{\text{i}}$$ (2)

The operating costs *Z*_*3*_ of second and third level cold chain storage facilities are related to their location, type, and capacity.


$$Z_{3}=\sum_{i=1}^{P_{n}}\sum_{n=1}^{N}\sum_{l\in L}\sum_{h\in H}O_{nlh}^{i}\;Cap_{nl\hbar }^{i}$$ (3)

3. Transportation cost

The calculation of transportation cost mainly depends on the price, distance and quantity of transportation, so the cost *Z*_*4*_ is:

$$Z_{4}=\sum_{n=2}^{N}\sum_{i=1}^{P_{n}}\sum_{j=1}^{P_{n-1}}Y_{n}^{ij}\;S_{n}^{ij}D_{n}^{ij}M_{n-1}^{i}$$ (4)

4. Loss cost

The loss cost *Z*_*5*_ of fresh agricultural products includes two parts: the loss cost from the first level node to the second level node and the loss cost from the second level node to the third level node.


$$Z_{5}=\sum_{n=2}^{N}\sum_{s\in S}\sum_{i=1}^{P_{n}}\sum_{j=1}^{P_{n-1}}\alpha_{1}\left(e^{\beta_{1}D_{n}^{ij}/V}-1\right)M_{1}^{i}+\sum_{n=2}^{N}\sum_{s\in S}\sum_{i=1}^{P_{n}}\sum_{j=1}^{P_{n-1}}\alpha_{2}\left(e^{\beta_{2}D_{n+1}^{ij}/V^{\prime }}-1\right)M_{2}^{i}$$ (5)

#### 3.3.2 Constraint analysis

After considering the characteristics of production and circulation of fresh agricultural products and the purpose of construction of cold chain storage facilities in producing areas, the following constraints are set for the model.

1. Fresh agricultural products have requirements for the growth environment, and due to the influence of industrial agglomeration, the agricultural products produced in one place are often identical or similar, so only one type of cold chain storage facility is set up at an alternative node.
2. From the perspective of meeting demand and controlling costs, the number of cold chain storage facilities at each level should not be too much, and should not exceed the number of alternative nodes.
3. Except for a few large-scale production, most fresh agricultural products need to be shipped out through cold chain storage facilities at all levels, so the amount of goods at the upper level of storage facilities is affected by the amount of goods transported by the next level.
4. Due to the uncertainty of agricultural product sales, the quantity of circulation through different channels is not invariable. In order to maintain the stability of the model, it is assumed that the agricultural products of each cold chain storage facility are transported to its upper node.
5. Cold chain storage facilities are places where agricultural products gather, which can be shipped and should be shipped only after construction is completed.
6. Cold chain storage facilities are important nodes in the origin logistics of agricultural products, and once built, they should receive goods to better preserve and transport agricultural products.
7. In origin logistics, cold chain storage facilities are storage and transfer nodes of agricultural products, overall, the inflow of goods at each level of storage facilities should be the same as the outflow.
8. Cold chain storage facilities need to be built only when there is demand, so the first level of cold chain storage facilities should have agricultural products that need to be transported.

#### 3.3.3 Construction of the model

In summary, the objective function is the sum of construction cost, operating cost, transportation cost, and loss cost, calculated as follow:

$$\mathrm{Min}Z=Z_{1}+Z_{2}+Z_{3}+Z_{4}+Z_{5}$$ (6)

The model includes the following constraints.


$$\sum_{n=2}^{N}\sum_{l\in L}\sum_{h\in H}X_{nlh}^{i}\leq 1;\forall i$$ (7)

$$\sum_{i=1}^{P_{n}}X_{nlh}^{i}\leq G_{n};n=1,2,\ldots ,N;\forall l,h$$ (8)

$$M_{n}^{i}=\sum_{j=1}^{P_{n-1}}Y_{n}^{ij}M_{n-1}^{j}e^{-\partial T_{n}^{ij}};i=1,2,\ldots ,P_{n};n=2,3,\ldots N$$ (9)

$$\sum_{i=1}^{P_{n}}Y_{n}^{ij}=1;j=1,2,\ldots ,P_{n-1};n=2,\ldots ,N$$ (10)

$$\sum_{i=1}^{P_{n}}Y_{n}^{ij}-X_{(n-1)lh}^{j}=0;j=1,2,\ldots ,P_{n-1};n=2,3,\ldots ,N;\forall l,h$$ (11)

$$\left(X_{nlh}^{i}-0.5\right)\left(\sum_{j=1}^{P_{n-1}}Y_{n}^{ij}-0.5\right)>0;i=1,2,\ldots ,P_{n},n=2,\ldots ,N;\forall l,h$$ (12)

$$\sum_{i=1}^{P_{n}}\sum_{j=1}^{P_{n-1}}Y_{n}^{ij}\;M_{n-1}^{j}e^{-\partial T_{n}^{ij}}=\sum_{i=1}^{P_{n}}M_{n}^{i};n=2,\ldots ,N$$ (13)

$$M_{1}^{i}>0;i=1,2,3,\ldots ,P_{1}$$ (14)

The formula (7) in the constraints indicates that only one type of cold chain storage facility with a certain capacity can be built at each alternative node. (8) represents that the number of selected cold chain storage facility nodes does not exceed the number of nodes planned for construction at that level. (9) indicates that in addition to the first-level nodes, the volume of goods at all levels of cold chain storage facility nodes is determined by the volume transported by the lower-level nodes. (10) means that each cold chain storage facility node can only transport agricultural products to one upper node. (11) indicates that the selected cold chain storage facility node can and must transport agricultural products. (12) indicates that agricultural products must be received after the construction of cold chain storage facilities. (13) represents that starting from the second level of cold chain storage facilities, the total amount of goods received at each level is equal to the amount of goods that need to be transported at that level. (14) indicates that the quantity of goods to be transported at the first level cold chain storage facility point is greater than 0.

### 3.4 Model solution algorithm

#### 3.4.1 Algorithm selection

The CELP model of multi-stage cold chain storage facility layout optimization established above is a Mixed Integer Linear Programming (MILP) model, which belongs to NP-hard problem, and the common solving methods include precise algorithm and heuristic algorithm. The precision algorithm is suitable for small scale problems, and as the scale increases, the calculation time and memory of the precision algorithm increase exponentially, which makes it difficult to deal with large-scale problems in practical applications. Heuristic algorithm is fast, but can not guarantee the quality of solution when facing complex problems. This study is to solve a large-scale combinatorial optimization problem, so the applicability of these methods is not strong. The genetic algorithm (GA) is suitable for complex problems, especially those with very large solution space, and can find high-quality solutions close to the optimal solution in a short time. Moreover, some studies [23–25] have proved the effectiveness of applying GA to solve CFLP, so this paper also adopts GA to solve the established model.

#### 3.4.2 Solution steps

**Fig 3:**
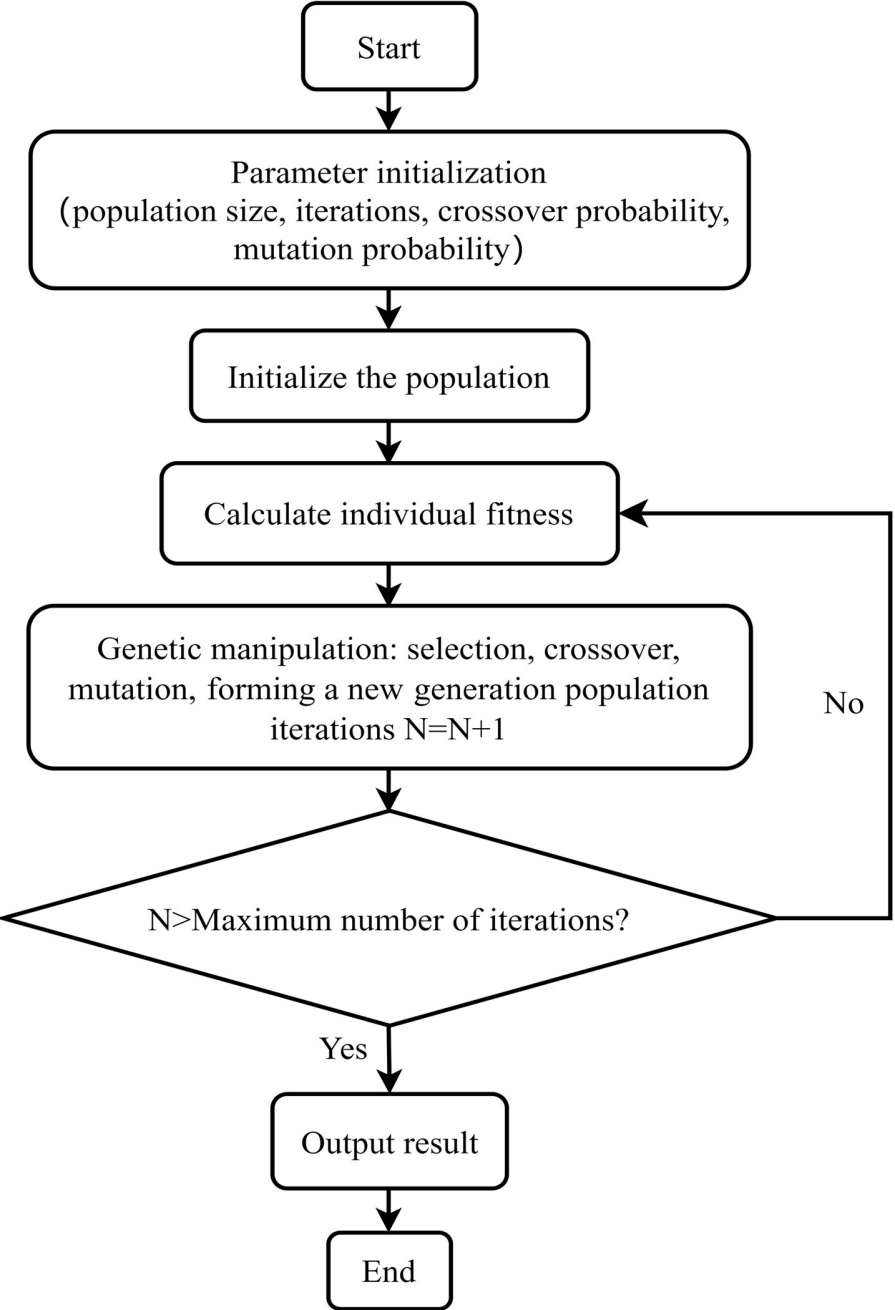
Genetic algorithm flowchart.

Genetic algorithm includes parameter initialization, construction of initial population, calculation of population fitness and genetic manipulation, etc. The main process is shown in Fig 3.

**1**. **Chromosome coding scheme**

By analyzing the layout optimization problem of multi-level cold chain storage facilities in rural areas, this paper presents the decision variable of the problem as a one-dimensional array by means of natural number coding.

**Fig 4:**
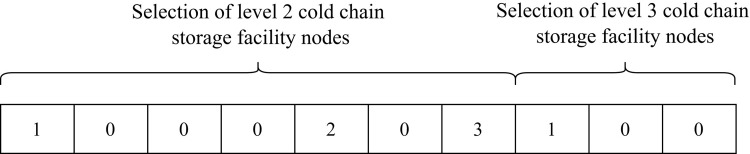
Example of chromosomal coding for cold chain storage facility node selection.

The chromosome coding for the selection of cold chain storage facility nodes is shown in Fig 4. The values of the selection status of the second level node are 0, 1, 2,3, where 0 indicates that the node is not selected, 1, 2, 3 indicates that the node is selected but belongs to different cold storage types. The value of the selection status of the third level node is 0 or 1, and at least one value is set to be 1 to represent the selected node.

**Fig 5:**
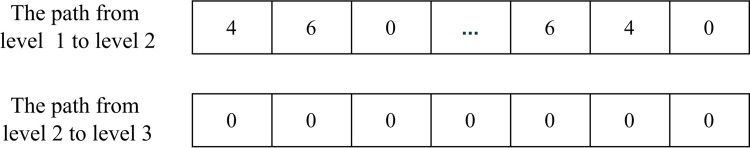
Example of chromosome coding for the path of cold chain storage facilities.

The chromosome coding for the transport path of cold chain storage facility nodes is shown in Fig 5. The path from level 1 to level 2 represents the agricultural products of the first level node are transported to the second level node, whose value is the index of the selected second level node. The path from level 2 to level 3 indicates that the agricultural products of the second level node are transported to the third level node, whose value is the index of the selected third level node.

The coding scheme effectively represents the entire transportation chain from the first level node of the cold chain storage facility to the second level node and then to the third level node, ensuring the rationality of the selection of each node and the transportation path to meet the actual needs of the logistics network.

**2**. **Initial group determination**

In the layout optimization of multi-level cold chain storage facilities in agricultural producing areas, the structure of individuals is first defined by chromosome coding, and then an initial population is randomly generated based on the set population size. In order to ensure the breadth of the search and avoid falling into local optimal solutions, it is necessary to ensure that the generated initial population has diversity. To avoid the presence of the same or similar individuals in the initial population, Hamming distances are used as a measure of differences between individuals. During initialization, the Hamming distance between all individuals is calculated and a threshold is set, and when the Hamming distance between two individuals is lower than this threshold, the two individuals are considered to be similar. If individuals whose similarity exceeds a threshold are detected, measures are taken to replace or adjust these individuals to maintain the diversity of the population. Next, based on the capacity limit of the cold chain logistics system, the transportation volume between each cold chain storage facility node is constrained, so as to generate a feasible solution that meet the conditions. In addition, for each generated individual, ensure that its path selection section only includes the selected nodes for optimization, in order to exclude any invalid paths and ensure the effectiveness and practicality of the solution.

**3**. **Design of fitness function**

The fitness function is used to evaluate the quality of each individual, calculate the total cost, and check the constraints. This paper obtains the total cost by calculating the construction cost, operating cost, transportation cost, and loss cost. And establish punishment mechanisms for individuals who do not meet the constraints to ensure that they are eliminated in the selection process. In genetic algorithm, the larger the fitness function value, the closer the algorithm is to the optimal value, so the negative of the model objective function is defined as the fitness function in this paper.

**4**. **Selection operation**

The first step in selection operation is to calculate the fitness of each individual. The fitness value indicates how good an individual is in the current problem, and the higher the value, the better the individual is. Then the fitness value is normalized to obtain the probability of each individual being selected. Finally, a random selection function is used to randomly select individuals from the current population according to the probability distribution of the previous step. The selected individuals will form the parent generation of the next generation for subsequent crossover and mutation operations. Individuals with high fitness are more likely to be selected, but individuals with low fitness also have a certain chance to be selected. This method can effectively avoid premature convergence to the local optimal solution.

**5**. **Crossover operator**

**Fig 6:**
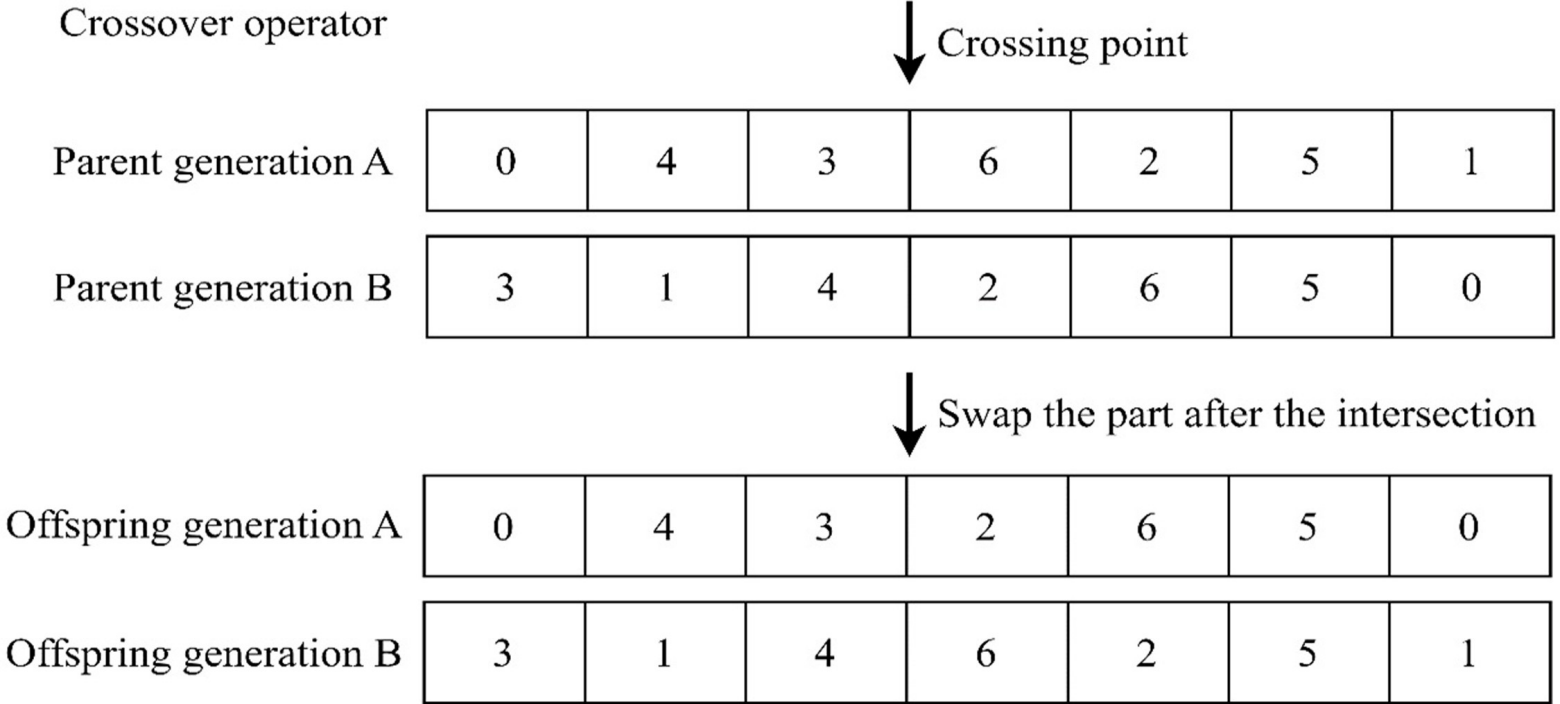
Cross operation.

Crossover is the replacement and recombination of fragments in the same position of two parent individuals to form a new individual. Crossover can improve the searching ability of genetic algorithm. The crossover probability is usually chosen at a higher value, about 80%-85%. In this paper, each part of the chromosome is crossed at a single point. For example, for a pair of paternal individuals Parent1 and Parent2, a crossing point is randomly generated at the same position of the chromosome, and this crossing point divides the individual gene sequence into two parts. In the section after the crossing point, the genetic sequences of the two parent individuals are exchanged to produce two new offspring individuals, an operation that can introduce diversity in the offspring, as shown in the Fig 6.

**6**. **Mutation operator**

**Fig 7:**
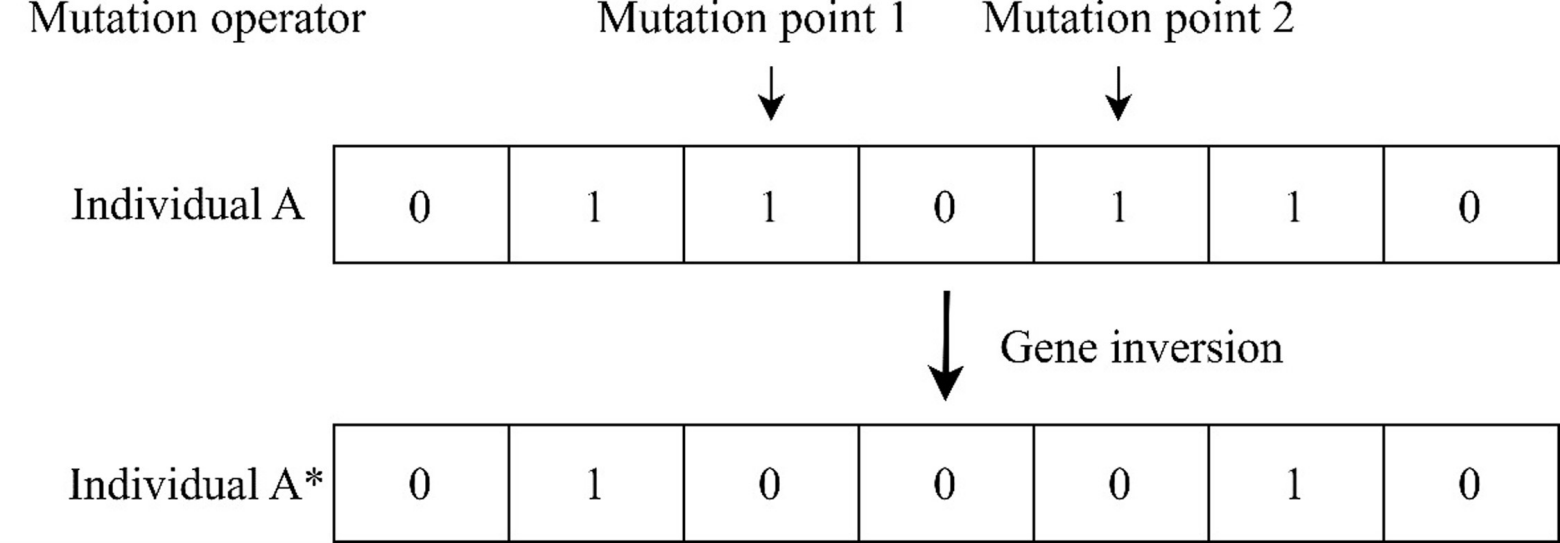
Mutation operation.

Mutation refers to the change of some gene values of individuals in the population, and the probability of mutation is usually selected to be very small, about 0.5% to 1%. In this paper, basic bit mutation is adopted, that is, the mutation point is randomly selected at the same position of chromosome to change the value of the gene at that point. If the mutation point falls on the selection section of the second or third level node, the gene will be flipped (from 0 to 1 or from 1 to 0), indicating that the corresponding node is selected or not selected, as shown in the Fig 7.

#### 3.4.3 Technical treatment

**1**. **Chromosome repair**

In order to maintain the integrity of the solution space, a repair mechanism is introduced, which can automatically correct the progeny individuals after crossing and mutation operations, so that they become valid solutions in the solution space again. At the same time, in order to ensure the feasibility of individual, constraint processing technology is used to ensure that all individuals meet the constraint conditions of the problem, including penalty function and repair operation. The penalty function is to punish the individual that does not satisfy the constraint by modifying the fitness function, so as to guide the genetic algorithm to develop in the direction of satisfying the constraint. The repair operation means that when an individual violates a constraint, a repair operation is performed to make it viable again.

**2**. **Algorithm convergence analysis**

In this paper, a genetic algorithm based on the elite individual retention mechanism is adopted. The time complexity of the algorithm is affected by the size of the initial problem, the setting of the model parameters, the size of the feasible solution space and the random generation of the initial solution. Gen [26] and He et al. [27] verified the convergence of genetic algorithm based on elite individual retention mechanism, which can ensure convergence to the global or local optimal solution of the model.

To determine when the algorithm should be stopped, the maximum number of iterations is set to the maximum limit at which the algorithm can run. When the algorithm reaches this number of iterations, the algorithm automatically stops regardless of whether it converges completely. Secondly, fitness curve is used to record the best fitness value of the current population in each iteration, and the trend graph is drawn as the number of iterations increased. If the optimal fitness value gradually increases with the increase of the number of iterations and eventually flattens out, it indicates that the algorithm is moving towards the optimal solution and eventually converges.

## 4 Example analysis

### 4.1 Example introduction

To verify the effectiveness of the multi-level cold chain storage facility layout model and algorithm, J County in China was selected as an example for analysis. In J County, vegetables are the main fresh agricultural products, covering root vegetables, leafy vegetables, and fruit vegetables. In 2023, the vegetable planting area reached 66400 hectares, with a yield of 207000 tons. However, cold chain storage facilities in J County are insufficient, and fresh agricultural products have a high loss rate and poor circulation. Therefore, it is very necessary to construct cold chain storage facilities reasonably to promote the circulation of agricultural products and the development of local economy.

### 4.2 Confirmation of alternative nodes

The county has 10 townships. According to the administrative division of J County, the cold chain storage facilities of agricultural products in the county can be arranged into three levels, of which the first level is the village level, the second level is the township level, and the third level is the county level.

1. Confirmation of county-level alternative nodes

The population and fiscal revenue of the townships in J County are shown in Table 3. It can be seen that the population size of towns 1, 2, 3 and 4 is larger and the economic situation is better. However, the transportation in Township 4 is relatively inconvenient. According to the situation of policy orientation, infrastructure utilization and market docking, the alternative sites of the third-level cold chain storage facilities in J County are selected as township 1, 2 and 3.

**Table 3:** Population and fiscal revenue (ten thousand yuan) of each township in J County.

Township number	1	2	3	4	5	6	7	8	9	10
Total population	109373	40397	35900	33600	11000	29300	31400	24500	30200	16200
Fiscal revenue	5370	3838	3715	3581	2435	2377	1870	1844	1450	1254

2. Confirmation of township-level alternative nodes

Township-level cold chain storage facilities are mainly to provide cold storage and transport services for fresh agricultural products in the town and nearby villages. Except for the 3 towns set as the third-level alternative nodes, the remaining 7 towns are all the second-level alternative nodes, that is, the township-level alternative nodes.

3. Confirmation of village-level nodes

Village-level storage facilities are the starting point of rural cold chain logistics, which is an important basis for determining the second and third level nodes. In this paper, the number and location of village-level cold chain storage facilities are determined by visiting local residents and conducting questionnaire surveys. The interviewees mainly included villagers and logistics practitioners in J County. The questionnaire questions are all single choice. If multiple or missed selections or five consecutive selections are the same, the questionnaire is invalid. In the survey, 100 electronic questionnaires and 130 paper questionnaires were sent out, and a total of 212 questionnaires were returned. After sorting out, it was found that 193 questionnaires were valid and 19 were invalid, with an effective recovery rate of 83.9%.

The survey results show that, 50.3% of the respondents were farmers, 37.1% were logistics practitioners, and 12.6% were other personnel. As for the problem of “the number of village-level cold chain storage facilities serving fresh agricultural products should be built in towns and villages”, the statistical results are shown in Table 4.

**Table 4:** Selection ratio of the number of cold chain storage facilities at village level (%).

Township number	Number of facilities			
	1–3	4–6	7–9	More than 10
1	23.2	12.3	56.7	7.8
2	21.3	40.6	22.5	15.6
3	48.3	18.4	30.6	2.7
4	27.2	53.2	11.0	8.6
5	60.3	18.3	12.6	8.8
6	20.4	56.6	18.3	4.7
7	26.3	48.3	13.7	11.7
8	45.3	30.6	14.4	9.7
9	23.6	46.7	19.5	10.2
10	39.4	36.3	20.6	3.7

According to the survey results,combined with the area of J County and the distribution of villages, with a service radius of no more than 4 kilometers as the basis, a total of 40 first-level cold chain storage facilities will be set up. The number of first-level nodes in each township is shown in Table 5.

**Table 5:** Number of first level cold chain storage facilities in each township.

Township number	1	2	3	4	5	6	7	8	9	10
Number of first-level nodes	7	4	3	4	3	5	4	3	4	3

Based on the above analysis, 50 nodes are involved in the layout decision of cold chain storage facilities in J County, including 40 first-level nodes, 7 second-level alternative nodes (renumbered) and 3 third-level alternative nodes.

### 4.3 Solution results

Considering the refrigeration needs of local vegetables, three types of cold storage were selected, that is, ventilated cold storage, air-cooled cold storage, and water-cooled cold storage, with capacities divided into three levels (m^3^), namely *W*_1_ = 300, *W*_2_ = 1000, *W*_3_ = 3000. According to the investigation of the local actual situation, combined with the research of Zhang [28] and Li [29], the construction and operation costs of cold chain storage facilities of different types and capacity levels are shown in Table 6. The actual distance (km) between all cold chain storage facility nodes is calculated based on their latitude and longitude. At the same time, the speed of the delivery vehicle is set at 40km/h. The transportation cost of refrigerated trucks from one cold chain storage facility to another is 0.25 yuan/ km·m^3^, and the loss cost coefficient of fresh agricultural products is *α*_1_ = 2, *α*_2_ = 1.5, *β*_1_ = 1.2, *β*_2_ = 1.

**Table 6:** Construction and operation costs of cold chain storage facilities.

Capacity level	Daily fixed construction cost (yuan)			Daily average unit variable construction cost (yuan/ m^3^)			Unit operating cost (yuan/ m^3^)		
	Ventilated cold storage	Air-cooled cold storage	Water-cooled cold storage	Ventilated cold storage	Air-cooled cold storage	Water-cooled cold storage	Ventilated cold storage	Air-cooled cold storage	Water-cooled cold storage
Small	400	300	100	1.5	1.25	0.75	2.4	2	1.2
Medium	1000	800	300	1.2	1	0.6	2	1.5	0.8
Large	1600	1350	600	0.9	0.75	0.3	1.6	1	0.4

The solution is based on the above data. In the process of model solving, the program iterated 500 times each time, and ran the solution program repeatedly 10 times. The running results are shown in Table 7.

**Table 7:** Statistics of algorithm operation results.

Order of results	Objective function value(yuan)	Selected nodes	
		Level 2	Level 3
1	8114.33	1, 2, 3, 5, 7	1, 3
2	8479.09	1, 4, 5, 7	2, 3
3	7141.60	1, 2, 5	3
4	6522.15	5, 6, 7	1
5	5809.42	1, 5, 7	1
6	6908.99	2, 5, 7	1
7	6790.01	1, 2, 5, 7	1
8	7581.17	2, 3, 5, 7	1
9	6474.46	1, 5, 6, 7	1
10	6522.15	5, 6, 7	1

**Fig 8:**
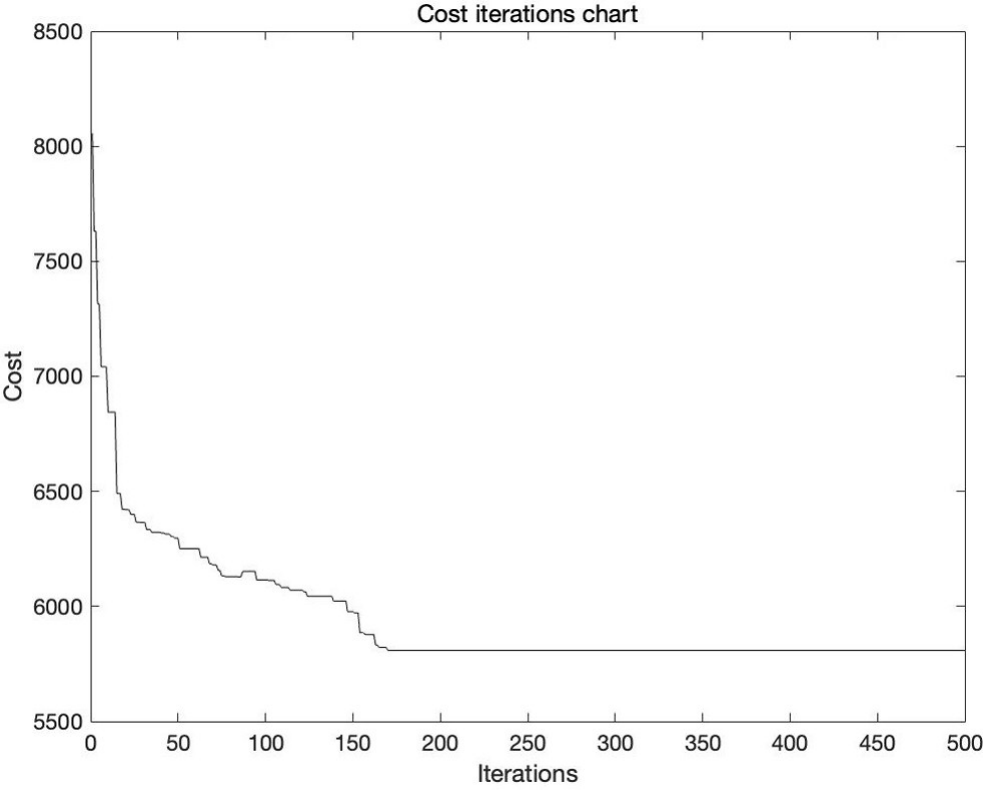
Convergence iteration of the algorithm.

From Table 7, it can be seen that the optimal solution is the result of the fifth solution. According to the convergence iteration of the fifth solution (Fig 8), in 500 iterations, when the number of iterations exceeds 200, the cost fluctuation has basically flattened out. Therefore, it can be considered that the setting of iteration times is set within a reasonable range, and the iteration results have reference significance.

**Fig 9:**
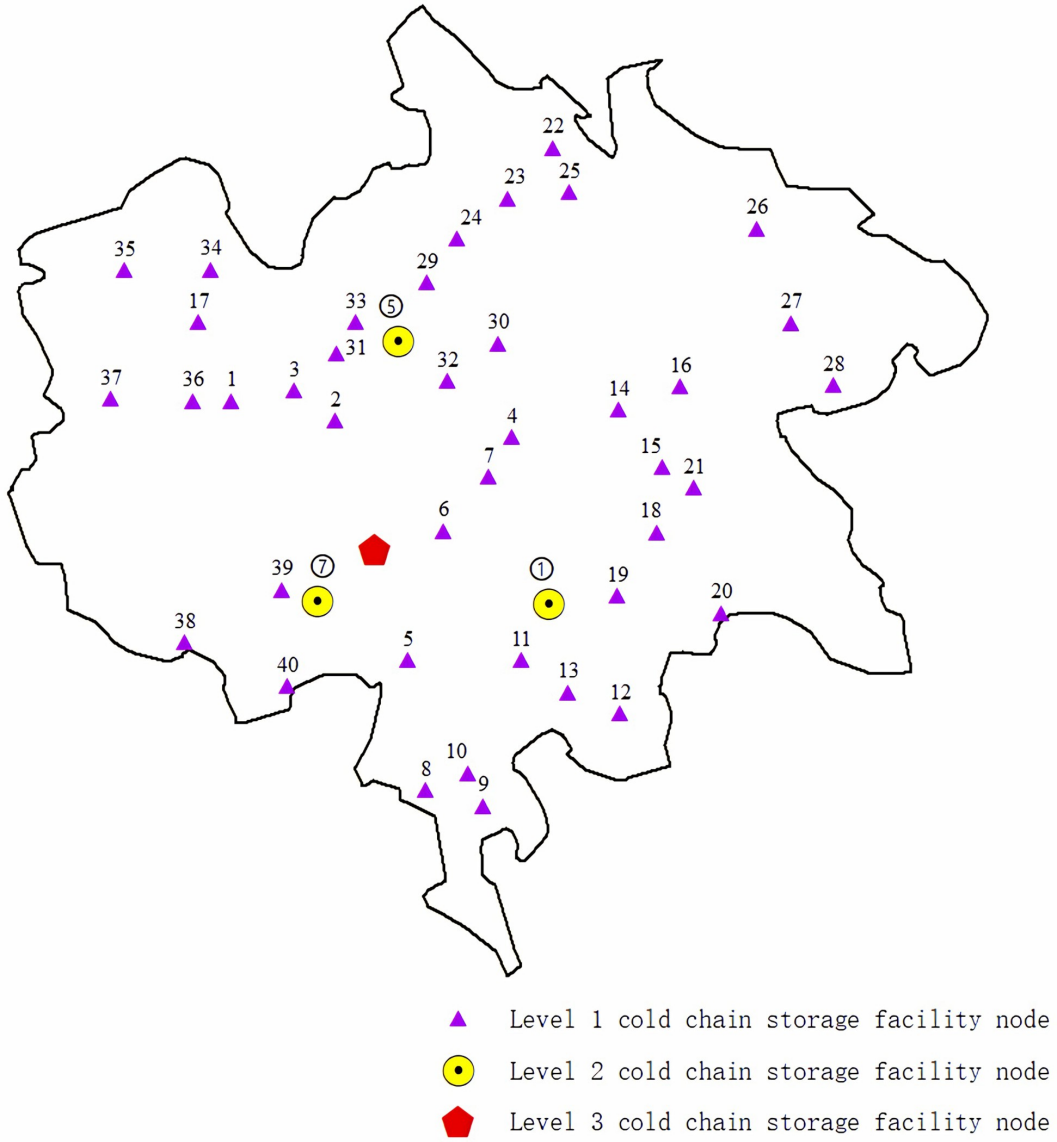
Optimized layout of multi-level cold chain storage facility nodes.

According to the actual situation, the lowest cost is 5809.42 yuan. The nodes of the second-level cold chain storage facilities are 1, 5 and 7, and the refrigeration methods are ventilated cold storage, air-cooled cold storage and water-cooled cold storage, respectively, with a capacity of 100 m^3^. A third level cold chain storage facility is built at alternative node 1 with a capacity of 500 m^3^. Table 8 shows the interconnection between nodes. And the optimized multi-stage cold chain storage facility layout is shown in Fig 9.

**Table 8:** Basic situation of cold chain storage facilities at all levels.

First level node	Second level node			Third level node	
number	number	Type	Capacity (m^3^)	number	Capacity (m^3^)
5, 10, 11, 13, 17, 19, 20, 28, 30, 34	1	Ventilated cold storage	1000	1	3000
1, 9, 14, 15, 16, 21, 22, 23, 24, 25, 26, 27, 29, 32, 33, 35, 37	5	Air-cooled cold storage	1000		
2, 3, 4, 6, 7, 8, 12, 18, 31, 36, 38, 39, 40	7	Water-cooled cold storage	1000		

From the example, it can be seen that the optimal solution can be obtained by bringing the real data into the model and applying the corresponding solution method, which can provide decision-making reference for the layout of cold chain storage facilities in agricultural producing areas. Through the application of the model, the number, location, type and capacity of cold chain storage facilities can be determined to better serve the circulation of fresh agricultural products and contribute to local economic prosperity.

## 5 Discussion

Based on the reality of China’s agricultural production and circulation, this paper studies the rationalization of the layout of cold chain storage facilities in producing areas. From the results of model construction and case analysis, it can be seen:

(1) It is necessary and feasible to layout multi-level cold chain storage facilities in agricultural production areas considering the type and capacity. The cold-chain storage facility layout decision-making model can be established by comprehensively considering the difference in demand for refrigeration of different agricultural products, the relationship between capacity and cost, and multi-level facility construction, etc. At the same time, optimization algorithms can be applied to quickly find a reasonable construction plan for cold chain storage facilities in producing areas. This is consistent with the results of Ma et al. [6] and He et al. [19]. The difference is that this paper considers many factors such as type, capacity and multilevel at the same time, so it has more comprehensive consideration of practical problems and higher application value. Through practical examples, this paper proves that the constructed model can help the layout of cold chain storage facilities in rural areas. However, due to the lack of existing cold chain resources in the region involved in the case, and there is no relevant planning scheme for the layout of cold chain storage facilities, this study fails to effectively compare the layout results with other schemes. But in any case, it provides a way of thinking for the construction of cold chain storage facilities for agricultural products in rural areas.

(2) Cost is an important factor that cannot be ignored in the construction of cold chain storage facilities in agricultural production areas. Similar to literature [5–8], this paper takes the pursuit of comprehensive cost minimization as the goal of cold chain storage facility layout decision, so as to reduce the waste of resources. Through the cost analysis, the construction cost, operation cost, transportation cost and loss cost are considered comprehensively, which is conducive to improving the effectiveness of decision-making. As the actual examples in this study involve a small area, which belongs to a small county in China, there are not many cold chain storage facilities that need to be laid out according to the optimization results. Through sensitivity analysis, it is found that the change of construction cost and other parameters has no obvious influence on the layout optimization scheme. However, according to the research from Ma et al. [6], when a large regional scope is involved, the loss cost coefficient of fresh agricultural products, fixed construction cost and unit variable construction cost all have an impact on the layout scheme of the precooling station. To some extent, the model established in this paper is more suitable for decision-making in a relatively large regional scope.

(3) Genetic algorithm is an effective method to solve the decision model of multi-level cold chain storage facility layout in agricultural produce areas. In this study, by comparing the methods of solving mathematical models, genetic algorithm is selected to solve the model. From the design of the algorithm and the application examples, the algorithm can explain the decision-making idea of the model and help to find the optimal solution quickly, which is an effective solution. The research results of literature [23–25] are again proved that genetic algorithm is conducive to solving the location problem with capacity constraints. Of course, if the model is further optimized, genetic algorithms and other methods can be mixed to improve the ability to solve problems.

However, there are some limitations to this study. It is based on the actual situation in China, where the proportion of small agricultural production is relatively large. Affected by the small scale of sales, most agricultural products need to flow through village, town, county, and then to cities. At the same time, considering the different circulation paths of agricultural products will greatly increase the complexity of the model and affect the stability, while the flux of agricultural products in different paths has great uncertainties. Therefore, this paper does not consider the case of allowing cross-level transportation when constructing the model. This will affect the effectiveness of the model to some extent, and may only get the near optimal scheme, but not the optimal scheme, when applied in practice. Since there is indeed a phenomenon of cross-level transportation in reality, especially for some large-scale agricultural produce places, agricultural products can be directly sent from the pre-cooling station to the sales place. Therefore, the case of allowing cross-level transportation can be considered in future research, in which the ratio of various types of cross-level transportation can be obtained by means of demand forecasting, in order to improve the effectiveness and application value of the model.

## 6 Conclusions

Based on the current situation and characteristics of fresh agricultural products circulation in rural areas of China, this paper discusses the optimization method of cold chain storage facilities layout in agricultural producing areas. In this paper, considering the cold storage needs of different fresh agricultural products and the scale economies of construction and operation of various cold chain storage facilities, a multi-level cold chain storage facility layout optimization model is established to determine the number, location, type and capacity of cold chain storage facilities at the same time. Genetic algorithm is used to solve the problem, and the effectiveness of the model and algorithm is verified by an example analysis.

The layout of cold chain storage facilities is of great significance to the circulation of fresh agricultural products in rural areas. In the process of modeling, this paper only considers the demand of fresh agricultural products transported from the countryside to the city, and does not consider the demand of goods transported from the city to the countryside. Further research can integrate the two types of cold chain needs and optimize the layout of cold chain storage facilities more comprehensively, so as to enhance the economic and social benefits of cold chain logistics in rural areas.

## Supporting information

S1 Appendix (DOCX)

S1 Data (DOCX)

S1 Graphical abstract (TIF)

## Abbreviations

FLP: Facility location problem
UFLP: Uncapacitated facility location problem
CFLP: Capacitated facility location problem
MILP: Mixed integer linear programming
NP: Non-deterministic polynomial
GA: Genetic algorithm
