# Supplementary material for: Layout optimization of multi-level cold chain storage facilities in agricultural producing areas considering type and capacity constraints
Source: PLoS One. 2025 Feb 11;20(2):e0313062. doi: 10.1371/journal.pone.0313062 (PMC11813114; doi:10.1371/journal.pone.0313062)
Supplement: S1 Appendix — (DOCX) [file pone.0313062.s001.docx]

**Appendix**

**Parameters and variables in the model.**

| Symbol | Definition |
| --- | --- |
| *Z* | Total daily cost. |
| $\text{G}_{\text{n}}$ | The number of nodes at level *n.* |
| $\text{P}_{\text{n}}$ | The number of alternative nodes at level *n*. |
| *N* | Level of nodes. |
| *S* | Type set of fresh agricultural products, *s*$\in$*S.* |
| *L* | Type set of nodes, *l*$\in$*L.* |
| *H* | Capacity level set of nodes, *h*$\in$*H.* |
| $\text{B}_{\text{nlh}}^{\text{i}}$ | Daily fixed construction costs for a storage facility of type *l* and capacity level *h* at node *i* of level *n.* |
| $\text{C}_{\text{nlh}}^{\text{i}}$ | Daily variable construction cost per unit for a storage facility of type *l* and capacity level *h* at node *i* of level *n.* |
| $\text{O}_{\text{nlh}}^{\text{i}}$ | Unit operating cost for a storage facility of type *l* and capacity level *h* at node *i* of level *n.* |
| $\text{S}_{\text{n}}^{\text{ij}}$ | The unit price of the product transported from the node *j* of level *n-1* to the node *i* of level *n.* |
| $\text{D}_{\text{n}}^{\text{ij}}$ | The distance from node *j* of level *n-1* to node *i* of level *n.* |
| $\text{M}_{\text{n}}^{\text{i}}$ | The daily quantity of products at node *i* of level *n.* |
| $\text{Cap}_{\text{nlh}}^{\text{i}}$ | The capacity of a storage facilities of type *l* and capacity level *h* established at the node *i* of level *n.* |
| $\text{T}_{\text{n}}^{\text{ij}}$ | The time from node *j* of level *n-1* to node *i* of level *n.* |
| *λ* | The operating cost per unit product of the node at the first level. |
| $\text{X}_{\text{nlh}}^{\text{i}}$ | Variable 0-1. When the candidate node *i* of level *n* is selected to build a storage facility of type *l* and capacity level *h*, $\text{X}_{\text{nlh}}^{\text{i}}$=1; otherwise $\text{X}_{\text{nlh}}^{\text{i}}$=0. |
| $\text{Y}_{\text{n}}^{\text{ij}}$ | Variable 0-1. When the agricultural products of node *i* of level *n* are supplied by node *j* of level *n*-1, $\text{Y}_{\text{n}}^{\text{ij}}$=1; otherwise, $\text{Y}_{\text{n}}^{\text{ij}}$=0. |
