# Supplementary material for: Layout optimization of multi-level cold chain storage facilities in agricultural producing areas considering type and capacity constraints
Source: PLoS One. 2025 Feb 11;20(2):e0313062. doi: 10.1371/journal.pone.0313062 (PMC11813114; doi:10.1371/journal.pone.0313062)
Supplement: S1 Data — (DOCX) [file pone.0313062.s002.docx]

**Data**

**1.** **Code link**

[**https://github.com/Dengjs2023/Nodes_siting**](https://github.com/Dengjs2023/Nodes_siting)

**2.** **Data used in the case study**

**Table 1. Distances between nodes of adjacent levels（km）.**

| Node number of level 2  Node number  of level 1 or 3 | | 1 | 2 | 3 | 4 | 5 | 6 | 7 |
| --- | --- | --- | --- | --- | --- | --- | --- | --- |
| Level 1 | 1 | 14.1 | 18.5 | 13.7 | 21.3 | 6.5 | 4.2 | 8.6 |
|  | 2 | 10.8 | 14.7 | 12.1 | 17.8 | 3.8 | 7.2 | 7.5 |
|  | 3 | 12.7 | 16.6 | 12 | 19 | 4.3 | 5.3 | 8.6 |
|  | 4 | 6.8 | 8.7 | 11.7 | 12.5 | 5.7 | 13.2 | 9.6 |
|  | 5 | 5.6 | 11.8 | 20.8 | 21.1 | 12.9 | 16.4 | 4.1 |
|  | 6 | 4.9 | 9.8 | 15.4 | 16.6 | 7.9 | 13.1 | 5.4 |
|  | 7 | 5.5 | 8.6 | 13.2 | 13.9 | 6.5 | 13.3 | 8.1 |
|  | 8 | 8.8 | 14.1 | 25.9 | 24.9 | 18.2 | 21.3 | 8.6 |
|  | 9 | 8.5 | 13.1 | 26.5 | 24.3 | 19.1 | 22.8 | 10.3 |
|  | 10 | 7.4 | 12.5 | 25.2 | 23.5 | 17.7 | 21.4 | 8.9 |
|  | 11 | 2.5 | 8.1 | 20.6 | 18.6 | 13.7 | 18.8 | 7.9 |
|  | 12 | 5.1 | 7.2 | 23.3 | 18.9 | 17.1 | 22.8 | 11.9 |
|  | 13 | 3.7 | 7.6 | 22.1 | 18.9 | 15.5 | 21 | 9.9 |
|  | 14 | 8.2 | 6.8 | 11.5 | 8.5 | 8.5 | 16.6 | 13.5 |
|  | 15 | 6.8 | 3.8 | 14.4 | 9.3 | 11 | 18.9 | 13.7 |
|  | 16 | 10 | 6.9 | 11.9 | 6.2 | 10.5 | 18.7 | 15.9 |
|  | 17 | 14.1 | 11.2 | 9.1 | 3.9 | 11 | 18.9 | 18.9 |
|  | 18 | 4.9 | 2.2 | 16.6 | 11.5 | 12.2 | 19.6 | 12.8 |
|  | 19 | 2.7 | 3.6 | 18.6 | 14.4 | 13.1 | 19.8 | 11.2 |
|  | 20 | 6.7 | 2.7 | 21 | 14.2 | 16.6 | 23.8 | 15.2 |
|  | 21 | 7.1 | 2.7 | 15.7 | 9.5 | 12.4 | 20.2 | 14.5 |
|  | 22 | 18.4 | 17.4 | 2.4 | 10.4 | 9.6 | 15.3 | 20.3 |
|  | 23 | 17 | 17 | 1.5 | 11.9 | 7 | 12.5 | 17.9 |
|  | 24 | 15.1 | 15.8 | 3.7 | 8.9 | 4.6 | 10.7 | 15.5 |
|  | 25 | 16.7 | 15.5 | 3.6 | 12.6 | 8.8 | 15.4 | 19 |
|  | 26 | 17.1 | 13.4 | 10.4 | 2.4 | 14 | 21.6 | 22.1 |
|  | 27 | 14.6 | 10.1 | 13 | 1.7 | 14.4 | 22.5 | 20.8 |
|  | 28 | 13.8 | 8.4 | 15.9 | 4.6 | 16.2 | 24.4 | 21 |
|  | 29 | 13.7 | 15.1 | 5.7 | 13.5 | 2.6 | 9.3 | 13.5 |
|  | 30 | 10.7 | 11.5 | 7.7 | 11.2 | 3.8 | 12 | 12.5 |
|  | 31 | 12.7 | 15.9 | 9.8 | 17.2 | 2.3 | 6.1 | 10 |
|  | 32 | 9.7 | 11.8 | 9.4 | 13.5 | 2.5 | 10.4 | 10.1 |
|  | 33 | 13.4 | 16 | 8.4 | 16.3 | 1.7 | 6.6 | 11.4 |
|  | 34 | 8.7 | 13.3 | 26.6 | 24.6 | 19.1 | 22.8 | 10.2 |
|  | 35 | 20.6 | 24.4 | 14.1 | 24.7 | 10.5 | 2.7 | 15.2 |
|  | 36 | 16.7 | 21.1 | 14.9 | 23.5 | 8.7 | 2.6 | 10.6 |
|  | 37 | 18.1 | 22.8 | 16.9 | 25.6 | 10.8 | 4.1 | 11.2 |
|  | 38 | 13.4 | 19.5 | 22.7 | 26.9 | 14.5 | 13.3 | 5.1 |
|  | 39 | 9.8 | 15.7 | 19.3 | 22.8 | 11 | 11.8 | 1.4 |
|  | 40 | 9.2 | 15.3 | 20.9 | 23.4 | 12.6 | 13.9 | 1.6 |
| Level 3 | 1 | 12 | 19 | 19 | 29 | 10 | 13 | 3.7 |
|  | 2 | 11 | 19 | 32 | 39 | 38 | 25 | 13 |
|  | 3 | 12 | 5.3 | 19 | 14 | 17 | 23 | 19 |

**Table 2. Average output of agricultural products at the node of the first level (m³).**

| Type  number  Node  number | 1 | 2 | 3 | 4 | 5 | 6 |
| --- | --- | --- | --- | --- | --- | --- |
| 1 | 0.4776 | 0.1299 | 0.1013 | 0.0085 | 1.4236 | 7.3095 |
| 2 | 0.5309 | 0.1129 | 0.2393 | 0.0040 | 1.3568 | 6.3283 |
| 3 | 0.0744 | 0.2266 | 0.1686 | 0.0004 | 2.0856 | 4.2526 |
| 4 | 0.5353 | 0.2353 | 0.1583 | 0.0147 | 2.5724 | 4.0817 |
| 5 | 0.3706 | 0.0553 | 0.2642 | 0.0192 | 2.4184 | 7.6946 |
| 6 | 0.0571 | 0.1450 | 0.0823 | 0.0334 | 1.9016 | 5.1868 |
| 7 | 0.1633 | 0.1319 | 0.2181 | 0.0068 | 1.2724 | 5.2533 |
| 8 | 0.3206 | 0.1913 | 0.2171 | 0.0320 | 1.5064 | 6.8506 |
| 9 | 0.5611 | 0.2100 | 0.1096 | 0.0208 | 0.6180 | 0.2598 |
| 10 | 0.5656 | 0.2233 | 0.1636 | 0.0296 | 1.1948 | 4.9049 |
| 11 | 0.0924 | 0.0817 | 0.0218 | 0.0346 | 2.5076 | 1.5558 |
| 12 | 0.5689 | 0.2011 | 0.0155 | 0.0730 | 0.2228 | 2.0406 |
| 13 | 0.5610 | 0.1939 | 0.1529 | 0.0146 | 0.6836 | 7.5398 |
| 14 | 0.2844 | 0.0481 | 0.2244 | 0.0211 | 0.6032 | 0.2439 |
| 15 | 0.4690 | 0.0352 | 0.2690 | 0.0117 | 1.2728 | 4.1666 |
| 16 | 0.0831 | 0.1475 | 0.0374 | 0.0109 | 1.6248 | 1.4282 |
| 17 | 0.2471 | 0.2841 | 0.1638 | 0.0696 | 1.7684 | 3.8596 |
| 18 | 0.5367 | 0.1007 | 0.1352 | 0.0464 | 1.0300 | 6.0615 |
| 19 | 0.4643 | 0.1732 | 0.0034 | 0.0441 | 0.9568 | 4.2565 |
| 20 | 0.5624 | 0.0663 | 0.0971 | 0.0116 | 0.0760 | 4.0066 |
| 21 | 0.3843 | 0.2224 | 0.0468 | 0.0683 | 0.0984 | 0.5071 |
| 22 | 0.0210 | 0.0755 | 0.2288 | 0.0498 | 0.3556 | 5.8001 |
| 23 | 0.4977 | 0.1498 | 0.0897 | 0.0281 | 1.8780 | 0.3608 |
| 24 | 0.5474 | 0.2069 | 0.1523 | 0.0411 | 0.2780 | 0.6077 |
| 25 | 0.3979 | 0.2636 | 0.0478 | 0.0322 | 0.2572 | 4.4366 |
| 26 | 0.4441 | 0.2840 | 0.1734 | 0.0061 | 0.6820 | 0.8227 |
| 27 | 0.4351 | 0.1620 | 0.0758 | 0.0192 | 0.8732 | 6.9583 |
| 28 | 0.2299 | 0.0410 | 0.1884 | 0.0099 | 1.7700 | 6.9532 |
| 29 | 0.3841 | 0.0442 | 0.1985 | 0.0621 | 2.3048 | 6.1443 |
| 30 | 0.1003 | 0.0763 | 0.2155 | 0.0655 | 2.3780 | 7.0193 |
| 31 | 0.4139 | 0.2489 | 0.1298 | 0.0696 | 2.0732 | 5.6099 |
| 32 | 0.0187 | 0.0753 | 0.0242 | 0.0771 | 1.7024 | 4.4106 |
| 33 | 0.1623 | 0.2410 | 0.0659 | 0.0641 | 1.2868 | 8.3237 |
| 34 | 0.0270 | 0.0721 | 0.2631 | 0.0089 | 2.0284 | 5.5197 |
| 35 | 0.0570 | 0.2750 | 0.0439 | 0.0393 | 1.8620 | 6.8067 |
| 36 | 0.4826 | 0.1035 | 0.2378 | 0.0392 | 2.3532 | 8.2752 |
| 37 | 0.4073 | 0.0582 | 0.1551 | 0.0271 | 2.3196 | 3.6775 |
| 38 | 0.1859 | 0.0743 | 0.2869 | 0.0721 | 0.8700 | 1.2746 |
| 39 | 0.5570 | 0.1823 | 0.0225 | 0.0723 | 1.8192 | 0.7099 |
| 40 | 0.0201 | 0.1401 | 0.1275 | 0.0756 | 0.5152 | 1.1326 |
