# Supplementary figures and images for: Layout optimization of multi-level cold chain storage facilities in agricultural producing areas considering type and capacity constraints
Source: PLoS One. 2025 Feb 11;20(2):e0313062. doi: 10.1371/journal.pone.0313062 (PMC11813114; doi:10.1371/journal.pone.0313062)

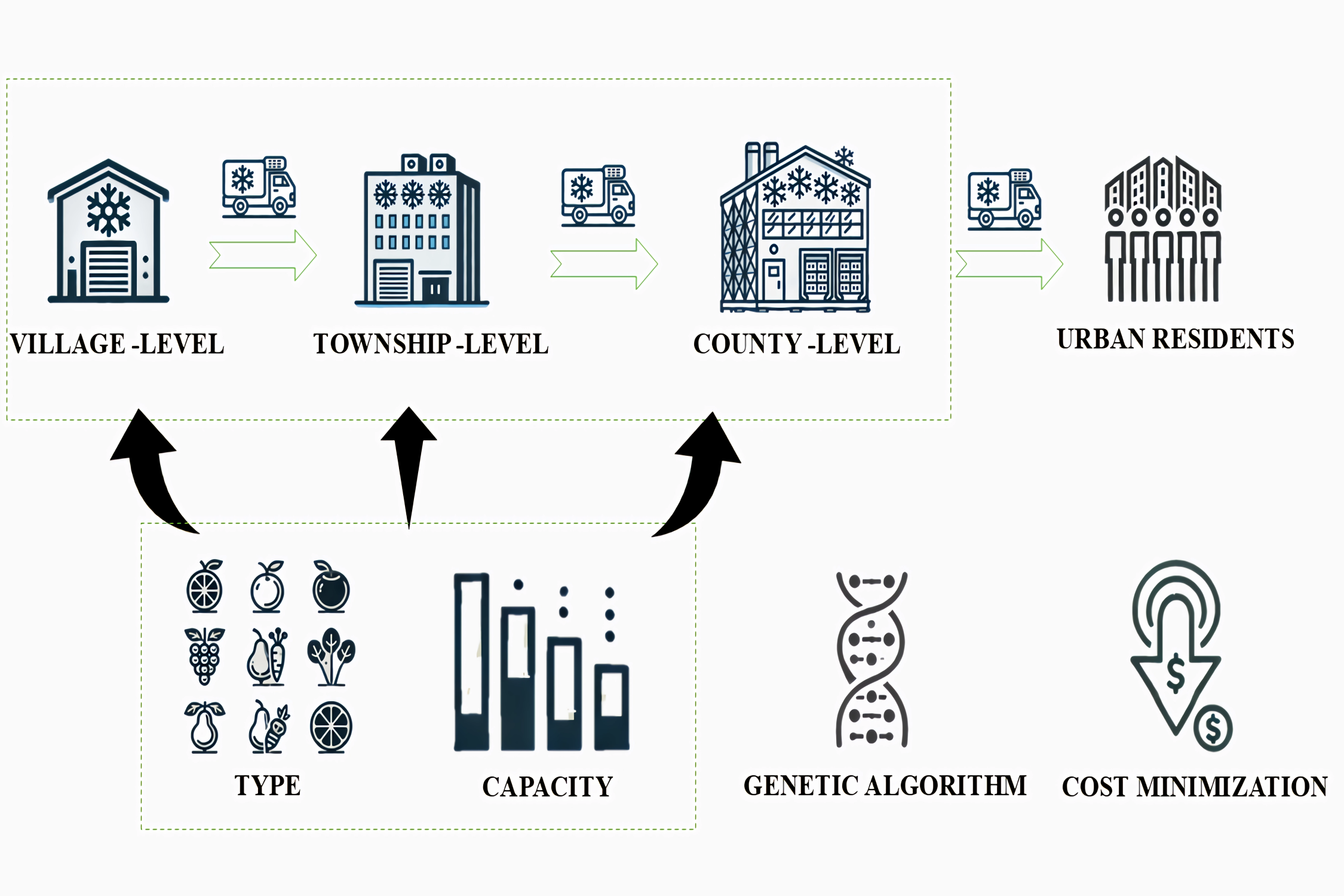

Supplement: S1 Graphical abstract — (TIF) [file pone.0313062.s003.tif]
